# Social risks and social needs in a health insurance exchange sample: a longitudinal evaluation of utilization

**DOI:** 10.1186/s12913-022-08740-6

**Published:** 2022-11-28

**Authors:** Cara C. Lewis, Salene M. W. Jones, Robert Wellman, Adam L. Sharp, Laura M. Gottlieb, Matthew P. Banegas, Emilia De Marchis, John F. Steiner

**Affiliations:** 1 Kaiser Permanente Washington Health Research Institute, 1730 Minor Ave Suite 1600, Seattle, WA 98101 USA; 2 Public Health Sciences Division, Fred Hutchinson Cancer Research Center, Seattle, WA USA; 3 Research and Evaluation Department, Kaiser Permanente Southern California, Pasadena, CA USA; 4 Health Systems Science Department, Kaiser Permanente School of Medicine, Pasadena, CA USA; 5 Center for Health and Community, University of California San Francisco, San Francisco, CA USA; 6 Social Intervention Research and Evaluation Network, University of California San Francisco, San Francisco, CA USA; 7 Department of Family and Community Medicine, University of California San Francisco, San Francisco, CA USA; 8 Kaiser Permanente Center for Health Research, Portland, OR USA; 9 Institute of Health Research, Kaiser Permanente, Denver, CO USA

**Keywords:** Social Risk, Social Needs, Health Care, Utilization, Longitudinal

## Abstract

**Background:**

Health systems are increasingly attempting to intervene on social adversity as a strategy to improve health care outcomes. To inform health system efforts to screen for social adversity, we sought to explore the stability of social risk and interest in assistance over time and to evaluate whether the social risk was associated with subsequent healthcare utilization.

**Methods:**

We surveyed Kaiser Permanente members receiving subsidies from the healthcare exchange in Southern California to assess their social risk and desire for assistance using the Accountable Health Communities instrument. A subset of initial respondents was randomized to be re-surveyed at either three or six months later.

**Results:**

A total of 228 participants completed the survey at both time points. Social risks were moderate to strongly stable across three and six months (Kappa range = .59-.89); however, social adversity profiles that included participants’ desire for assistance were more labile (3-month Kappa = .52; 95% CI = .41-.64 & 6-month Kappa = .48; 95% CI = .36-.6). Only housing-related social risks were associated with an increase in acute care (emergency, urgent care) six months after initial screening; no other associations between social risk and utilization were observed.

**Conclusions:**

This study suggests that screening for social risk may be appropriate at intervals of six months, or perhaps longer, but that assessing desire for assistance may need to occur more frequently. Housing risks were associated with increases in acute care. Health systems may need to engage in screening and referral to resources to improve overall care and ultimately patient total health.

## Background

Social determinants of health (SDoH) are defined as factors in a person’s life that affect their health and ability to engage in healthcare [1, 2]. Mounting research suggests SDoH are more accurate predictors of premature mortality than genetics or healthcare access [3–10]. As a result of the compelling evidence that social adversity significantly influences health and costs of care [11–16], health systems are increasingly attempting to intervene on social adversity as a strategy to improve health care outcomes. Health system activities often begin with screening patients for social risks (e.g., housing insecurity, food insecurity, financial strain). However, insufficient research exists to inform how frequently screening must occur to accurately capture peoples’ experience of social adversity. Few longitudinal studies of social risks exist in the absence of an intervention to identify patterns over time.

As social adversity screening has increased in health care settings, several studies have documented that patients who endorse social risk, such as the experience of financial strain, may in parallel decline related assistance from their healthcare system [17]. The reasons for this are likely varied and may include peoples’ priorities and competing demands, prior experience with social services, and prior experience with their healthcare system. It is unclear whether this phenomenon is unique to a subgroup of people, severity, or type of social risk domain, or if it reflects a transitional period wherein people may go on to endorse interest in assistance if the social risk persists. Ideally, health systems would help people address a given social risk before it has adverse effects on their health and wellness.

Screening results are often used to identify people who may benefit from additional services or referrals to community-based organizations [17]. However, these interventions can be difficult to staff without a better understanding of the stability of the social adversity experience. Moreover, a recent review of health system social risk interventions found that while most interventions improved relevant outcomes, few studies had even explored the impact on healthcare utilization [17]. Evidence from a recent randomized clinical trial of a 3-month social risk intervention did not reveal reductions in healthcare utilization [18]. One potential reason for the lack of impact on healthcare utilization could be that both social risk and patterns of healthcare use, are dynamic [19].

To address these gaps in the literature, this manuscript reports on a longitudinal survey of social risk in a subpopulation of US residents insured via the subsidized exchange in an integrated healthcare system. This manuscript was guided by three aims: (1) to examine the stability of social risk by key domains (e.g., transportation, housing, food security) over time; (2) to explore how people’s social risk and interest in assistance with social risk change in relation to one another over time; and (3) to evaluate if and how endorsement of social risk is associated with subsequent healthcare utilization. We anticipate that findings from this study could help inform national social care-related initiatives by shedding light on the ideal timing of repeated social health screening efforts, the types of social health interventions in which people may be most interested, and the business case to justify additional staffing needs to bolster these efforts.

## Method

### Participants

The study sample was recruited from 134,355 Kaiser Permanente (KP) Southern California members who obtained insurance coverage through the subsidized exchanges as of September 1, 2018. This population is an important, yet understudied group, who have income levels that preclude their eligibility from many governments and/or community financial assistance programs but are still low enough to experience financial strain and related social risks. The subsidized exchanges provided health insurance to people who met specific eligibility criteria, including income (between 100 and 400% of the Federal Poverty Level), ineligibility for Medicaid or other public health care programs, US residency status, and availability of health insurance coverage through their employer—a program that was created under the Affordable Care Act. Potential participants (*N* = 1,008) were randomly selected across age (18–26, 27–44, 45–61, 62 and over), gender, and language (English vs. Spanish) strata.

### Survey overview and fielding procedures

The survey development is described in more detail elsewhere [20], as are the fielding procedures [21]. To address stability in social risk, the survey was administered once and then repeated at either three or six month (participants were randomly assigned to follow up time frame) to explore differential change over two-time intervals that may reflect practical screening windows. Participants received an advance letter along with a $2 pre-incentive and an online web link to the survey. Participants received follow-up emails, phone calls, text messages, and if requested, a paper survey in the mail. Those who completed at least 50% of the survey received a $20 cash incentive. The study was part of ongoing quality improvement efforts. As such, it was deemed not human subjects research after administrative review by the KP International Review Board (IRB) (Human Subjects Assurance Number/Federal Wide Assurance Number: 00002344); informed consent was not required.

### Measures

#### Social Risks

Social risks were measured using items from the Accountable Health Communities (AHC) instrument developed by the Centers for Medicare and Medicaid Innovation [22]. The AHC social risk screening tool has six items assessing housing security, housing quality (e.g. mold, pests, smoke detectors not working), transportation difficulties, food insecurity (2 items), and trouble paying for utilities. This relatively newer tool has been utilized in a variety of settings and populations [22]. Only one study has explored evidence of psychometric strength [23] in which it demonstrated convergent validity with a similar tool used widely in KP [21].

#### Interest in Assistance with Social Risks

One survey item asked if participants desired assistance with the following social risks, an endorsement of which was considered a *social need*: housing, food access, transportation (medical and non-medical), and utilities. Participants could check multiple risk factors for which they wanted assistance, or they could check ‘none.’

#### Healthcare Utilization Outcomes

The use of healthcare was captured from electronic health records (EHR) and claims. Data were pulled for the 12 months before and the six months after the initial survey. Healthcare utilization outcomes included: primary care, specialty care, urgent care, and emergency department visits.

### Analytic strategy

#### Stability of Social Risk

For Aim 1 analyses, participants were coded as either having the risk or not having the risk at the initial and follow-up survey. These risks were then characterized as “stable,” “improving,” or “worsening” between these two survey points. We used the bias- and prevalence-adjusted Kappa statistic (PABAK) to assess the stability of reported social risk over time within each domain [24, 25]. Because roughly half of the sample completed each follow-up (3 months and 6 months), Kappa statistics were produced within each subsample (i.e., initial survey to 3 months and initial survey to 6 months). Kappa values can be interpreted as follows: No agreement = 0-0.20; Minimal = 0.21-0.39; Weak = 0.40-0.59; Moderate = 0.60-0.79; Strong = 0.80-0.90; Almost Perfect = above 0.90 [26].

#### Social Risk and Needs

For Aim 2 analyses, participants were categorized as having at least one social risk*,* if they endorsed any of the risk factors in any of the domains on the AHC screening tool. Participants were categorized as having a social need, if they wanted help with at least one social risk*;* otherwise, they were categorized as not having a need. Participants were categorized into four groups at both the initial survey time point and at follow-up: no social risk/need; at least one social risk/no need; no social risk/need assistance with at least one domain; and social risk/need. We used Cohen’s Kappa statistic to assess the stability of the social risk/need groups over time.

#### Utilization Outcomes

To assess which outcomes were prospectively associated with specific social risks, we conducted regression analyses with each healthcare utilization outcome as a dependent variable in separate models. Poisson regression models controlled for age, gender, race/ethnicity, the Neighborhood Deprivation Index [6], and language, as well as the value for the respective outcome in the 12 months before the survey. Each of the five social risks from the initial survey timepoint were entered as a dichotomous predictor of healthcare utilization in the six months following the survey. We also considered results with a Type I error correction. We used a Bonferroni correction across all 20 significance tests for the healthcare utilization outcomes resulting in an alpha level of 0.0025 for each test with the Type I error correction.

## Results

Out of 1008 subsidized exchange members invited to complete the survey, 442 completed at least part of the survey (45% response rate) for the initial timepoint, of which 355 were eligible for randomization to complete a follow-up survey at either 3 months or 6 months later. Two hundred and twenty-eight participants completed a follow-up survey; 190 surveys were fielded at 3 months for a total of 124 completed surveys (response rate = 65.26%), and 161 were fielded at 6 months for a total of 110 completed surveys (response rate = 68.32%). Sample characteristics are reported in Table 1. Consistent with the stratified design, the initial sample was roughly equally distributed in terms of age and gender, with diversity in terms of race and ethnicity.

**Table 1:** Sample characteristics at baseline

	Overall		3-Month Survey Subgroup		6-Month Survey Subgroup	
	N	(%)	N	(%)	N	(%)
Age						
18 to 26 years old	39	17.1	19	16.1	20	18.2
27 to 44 years old	62	27.2	34	28.8	28	25.5
45 to 61 years old	49	21.5	26	22.0	23	20.9
62 years old or older	78	34.2	39	33.1	39	35.5
Gender						
Female	128	56.1	68	57.6	60	54.6
Male	100	43.9	50	42.4	50	45.5
Race/Ethnicity						
Black, African-American	11	4.8	6	5.1	5	4.6
Hispanic	33	14.5	17	14.4	16	14.6
Missing	38	16.7	24	20.3	14	12.7
Other	36	15.8	18	15.3	18	16.4
White, Caucasian	110	48.2	53	44.9	57	51.8
Food insecurity						
No	173	75.9	87	73.7	86	78.2
Yes	55	24.1	31	26.3	24	21.8
Housing insecurity						
No	204	89.5	107	90.7	97	88.2
Yes	24	10.5	11	9.3	13	11.8
Housing conditions problem						
No	167	73.2	92	78.0	75	68.2
Yes	61	26.8	26	22.0	35	31.8
Transportation difficulties						
No	207	90.8	106	89.8	101	91.8
Yes	21	9.2	12	10.2	9	8.2
Trouble paying for utilities						
No	212	93.0	109	92.4	103	93.6
Yes	16	7.0	9	7.6	7	6.4
Any risk						
No	122	53.5	67	56.8	55	50.0
Yes	106	46.5	51	43.2	55	50.0
Need food assistance						
No	224	98.2	118	100.0	106	96.4
Yes	4	1.8	0	0.0	4	3.6
Need housing assistance						
No	212	93.0	115	97.5	97	88.2
Yes	16	7.0	3	2.5	13	11.8
Need transportation assistance						
No	217	95.2	113	95.8	104	94.6
Yes	11	4.8	5	4.2	6	5.5
Need utilities assistance						
No	207	90.8	109	92.4	98	89.1
Yes	21	9.2	9	7.6	12	10.9
Any need						
No	185	81.1	101	85.6	84	76.4
Yes	43	18.9	17	14.4	26	23.6

### Aim 1

**Fig. 1:**
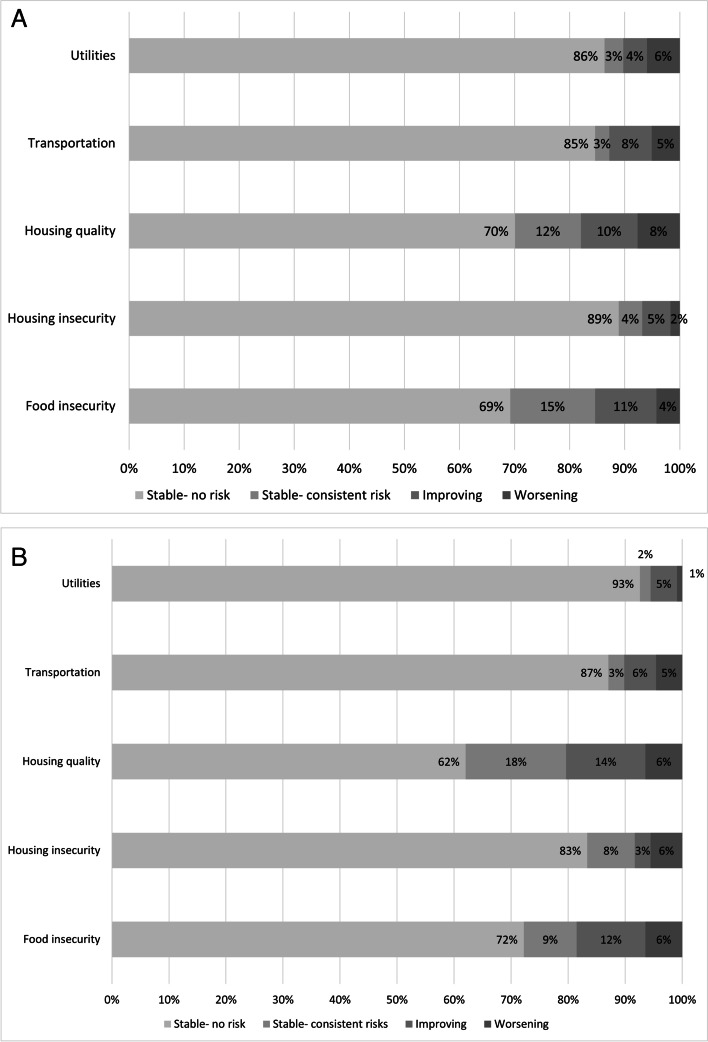
**a** Stability of Social Risk over 3 Months (*N* = 117). **b** Stability of Social Risk over 6 Months (*N* = 108) Improving means the participant reported the social risk at initial survey but not at the follow-up survey. Worsening means the participant did not report the social risk at the initial survey but reported it on the follow-up survey. Stable means the participant consistently reported no risk or the risk at both surveys

From initial survey to three months, housing insecurity was the most stable risk (Kappa = 0.86; 95% CI: 0.77, 0.95), followed by difficulty paying for utilities (Kappa = 0.79; 95% CI: 0.68, 0.90), transportation (Kappa = 0.74; 95% CI: 0.62, 0.86), food insecurity (Kappa = 0.69; 95% CI: 0.56, 0.82), and housing quality (Kappa = 0.64; 95% CI: 0.50, 0.78). Across domains, 4–11% of social risks improved whereas 2–8% worsened. From the initial survey to six months, risks were again moderately stable with some variability across domains with the order of prevalence in stable/consistent risk as follows: housing quality problem (Kappa = 0.59; 95% CI: 0.44, 0.74), food insecurity (Kappa = 0.63; 95% CI: 0.48, 0.78), transportation (Kappa = 0.80; 95% CI: 0.68, 0.91), housing insecurity (Kappa = 0.83; 95% CI: 0.73, 0.94), and paying for utilities (Kappa = 0.89; 95% CI: 0.80, 0.98). Across domains, 3–12% of social risks improved whereas 1–6% worsened. Figure 1a and b depict the percentage of respondents who improved, worsened, or remained consistent in their risk profile between the two surveys.

Due to the small sample size, we estimated precision post-hoc for the lowest and highest kappas on both the 3 and 6 months surveys using the R package kappa size [27]. For 3 months, the analysis for housing quality (kappa = 0.64) had an expected lower bound of 0.49 and the analysis for housing insecurity (kappa = 0.86) had an expected lower bound of 0.68. For 6 months, the analysis for housing quality (kappa = 0.59) had an expected lower bound of 0.44 and the analysis for utilities (kappa = 0.89) had an expected lower bound of 0.66. Given that studies should be ideally powered to detect a value greater than 0 such as 0.40 [25], these post-hoc analyses suggest the study was adequately sized to accurately estimate the kappas.

### Aim 2

**Fig. 2:**
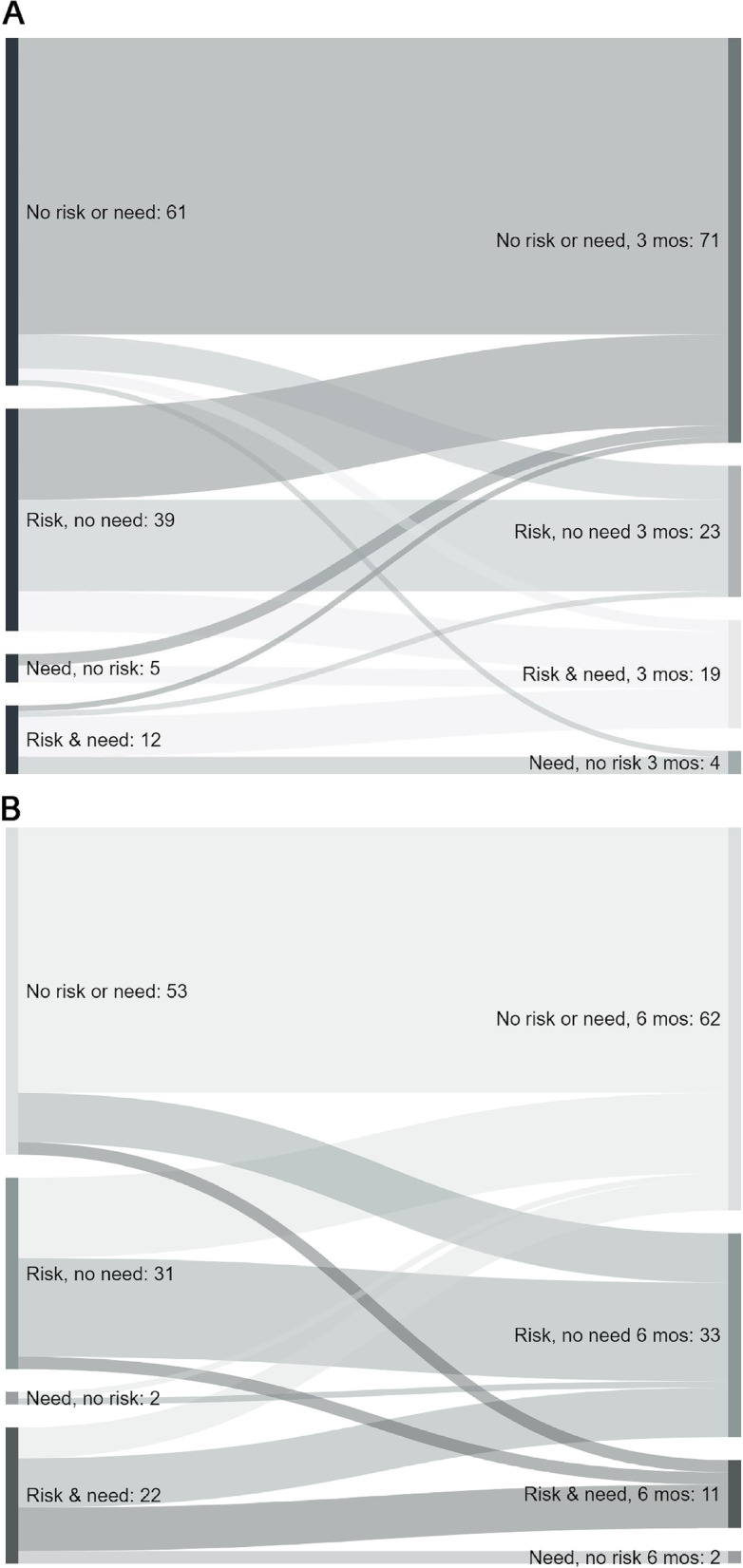
**a** Movement between Social Risk. Need Status from 3 month (*N* = 117). Only those participants who had social risk and need data at both the initial survey time point and 3 months later were included. Overall Kappa = .52; 95% CI: 0.41, 0.64. **b** Movement between Social Risk/Need Status from 6 months (*N* = 108). Only those participants who had social risk and need data at both the initial survey time point and 3 months later were included. Overall Kappa = .48; 95% CI: 0.36, 0.60

From initial survey to three (Kappa = 0.52; 95% CI: 0.41, 0.64) and six months (Kappa = 0.48; 95% CI: 0.36, 0.60), the social adversity profiles showed weak agreement; see Fig. 2a and b, respectively. The Sankey diagram depicts movement between risk profiles and indicates the greatest movement is toward risk resolution, from risk but no need to no risk or need. Only 50% of those with a risk and need at the initial survey (*n* = 22) reported a risk and need by six months (*n* = 11), with the majority still endorsing risk, but no longer desiring assistance, and several reporting complete resolution of risk by six months.

### Aim 3

Overall, results of Poisson regressions showed few social risks were associated with any utilization differences at 6 months (Table 2). Housing insecurity was associated with increases in ED visits (relative risk [RR] = 2.69, *p* = 0.01), and problems with housing quality was associated with more UC visits (RR = 1.61, *p* = 0.04), which do not hold when the Bonferroni correction is applied.

**Table 2:** Social risk and healthcare utilization 6 months later

	Primary Care, Outpatient Visits			Specialty Care, Outpatient Visits			Urgent Care Visits			ER Visits		
	RR	*P*-value	95% CI	RR	*P*-value	95% CI	RR	*P*-value	95% CI	RR	*P*-value	95% CI
Food insecurity	0.884	0.599	(0.559, 1.399)	0.642	0.182	(0.335, 1.231)	1.071	0.787	(0.652, 1.758)	0.978	0.962	(0.384, 2.489)
Housing insecurity	1.109	0.657	(0.702, 1.752)	1.361	0.353	(0.711, 2.608)	0.760	0.480	(0.355, 1.628)	**2.691**	**0.011**	(1.256, 5.763)
Housing condition	0.841	0.353	(0.585, 1.211)	1.254	0.365	(0.769, 2.044)	**1.608**	**0.038**	(1.027, 2.517)	1.367	0.436	(0.623, 2.996)
Transportation	1.057	0.807	(0.675, 1.657)	1.152	0.766	(0.455, 2.919)	0.440	0.103	(0.164, 1.181)	0.775	0.676	(0.235, 2.56)
Utilities	0.943	0.838	(0.537, 1.656)	**0.127**	**0.029**	(0.02, 0.81)	0.522	0.176	(0.204, 1.338)	0.596	0.465	(0.149, 2.387)

Analyses adjusted for age, gender, cost-sharing reduction subsidy, neighborhood deprivation index, race/ethnicity and the value of the outcome in the 12 months before the survey. Statistically significant results were no longer significant after type I error correction

## Discussion

This study examined the stability of social risks and needs over three or six months in a sample of people receiving subsidies from the healthcare exchange. Social risks were generally moderately to strongly stable over three months. Over six months, social risks were moderately to strongly stable except for housing quality which showed potentially weak to moderate stability. Social adversity profiles were showed weak stability over both three and six months; the greatest change observed across both time periods was that of risk resolution. Finally, when controlling for key demographic variables (e.g., gender, age, neighborhood deprivation) and prior utilization, in general, social risks were not related to utilization; however, we observed significant associations between housing insecurity and quality and increased use of expensive, emergent services six months later.

### Risk domain stability

Across individual social risks, most were stable over time with a minority either improving (risk resolving) or worsening (new risk reported). The proportion experiencing changes ranged from 15–20% (food insecurity, housing quality problems) to 6–11% (trouble paying for utilities and housing insecurity). These findings are largely consistent with the broader literature that suggests losing one’s housing is quite rare in the general population, and even rapid rehousing interventions can require up to 120–143 days for resolution [28, 29]. Trouble paying for utilities may be more stable for different reasons. Specifically, there is some literature to suggest that people prioritize paying for rent and food, and utilities are routinely deprioritized [30, 31]. However, trouble paying for utilities was rare in this sample perhaps because these individuals received healthcare subsidies, which may free up financial resources to pay for other material needs, or because respondents could access utility assistance benefits for which income eligibility requirements may be more generous than Medicaid and SNAP [32]. Conversely, food insecurity is notoriously labile [33, 34], given fluctuations in income and/or challenges managing fixed/limited incomes, a problem that has been exacerbated by the COVID-19 pandemic [35–37].

The general stability of social risk has implications for screening cadence and intervention evaluation design. Risks were mostly stable over three and six months, which suggests that screening every six months is likely sufficient to sensitively detect emergence or resolution of social risks in this population. This recommendation is consistent with several other studies [38], acknowledging that some scholars advocate for annual screening, which reduces the possibility of over-screening [39], may be more patient-centered, and improve health system feasibility. These data suggest that time to follow-up in intervention evaluations may appropriately be set to three or six months since social risks in this population appear relatively stable in the absence of intervention. However, this study is one of few longitudinal studies of social risk and some domains (e.g., housing quality) may be more naturally labile particularly among marginalized populations.

### Social adversity profile stability

Although the social adversity profiles were somewhat stable, many people did transition between groups from the initial survey to three or six months. For example, nearly half of those endorsing a risk but no need initially reported their risks resolved three months later, whereas far fewer of those initially in the “no risk and no need” group endorsed risk three months later and most in this group stayed free of social risk. This pattern was almost identical across six months of observation. Importantly, a portion of those endorsing risk but no need initially went on to desire assistance at three and six months; however, this portion was much smaller across the six-month timeframe. This may suggest that desire for assistance is more labile, and/or that individuals were able to connect with resources by six months, which we were unable to track in this initiative. In support of the former interpretation, as noted above, it is common in the literature to report on a phenomenon in which far fewer people who endorse social risk want help. Reasons for not wanting help from the healthcare system include stigma, privacy concerns, mistrust in the healthcare system, perception that this is not the role of the healthcare system, or concern about disclosing a social need for which the healthcare system cannot provide assistance [38, 40, 41]. To the latter point, it might be that social health interventions may take six months or more to resolve need, and perhaps longer to resolve the underlying social risk. However, much of this is speculation in absence of having a true baseline or intervention group for comparison.

These findings might inform social health screening and intervention initiatives. First, these data suggest that screening could focus initially on identifying social risk, as a measure of underlying social adversity, rather than identifying those with desire for assistance, which may be associated with psychological constructs such as readiness to change. This approach sets the stage for health systems to pair screening with social risk-informed care (or social risk adjusted care [42]) in which patients’ social context is considered carefully by physicians and care teams in collaboratively generating their care plan [43], consistent with precision medicine [44]. Although this is not (yet) standard practice, this approach would warrant screening for risk to ensure those realities inform shared decision making even for members who do not wish for health systems to target their social issues directly, and especially in cases where resources do not exist to resolve risks. Conversely, screening focused on desire for assistance with social risk presents an obvious starting place for health systems eager to connect patients with resources, but given its lability, may warrant more frequent assessment to be responsive to patients’ preferences.

### Social risk predicts utilization

Among members receiving health insurance subsidies, we observed two statistically significant prospective associations with utilization. Specifically, we found that housing insecurity was associated with increased use of emergency department visits (with an almost three-fold relative risk compared to those without housing insecurity) and housing condition problems predicted increased use of urgent care services. Housing interventions thus have the potential to decrease use of expensive emergent services [45–47]; however, it is important to note that we did not control for possible confounders such as serious persistent mental illness that may drive housing challenges and utilization issues. These findings should be considered preliminary.

### Limitations

Several limitations of this study should be considered. First, this was a small sample of people receiving health insurance subsidies on the healthcare exchange within a single large integrated, healthcare system in Southern California and, thus, the results might not generalize to other regions or subpopulations. Second, not all social risk domains were assessed, such as interpersonal violence. Third, the sample was not restricted to people struggling with homogeneous health conditions, which could dilute relations between social risk and utilization that are important to understand. Fourth, structural vulnerability [48], and systems of oppression, may lead to both social risk and high/low healthcare use, and we were unable to directly explore these influences. Fifth, several statistical models were run, which may elevate the risk of Type I error in reporting. Finally, we were not able to track whether this health system was systematically providing social risk interventions that could explain the cases of improvement observed.

## Conclusion

Overall, most social risks were stable over three or six months while desire for assistance was less stable. Given this, the question remains: How frequently should we be asking our patients about their social conditions? Until a business case for social needs targeted care is made, health systems will struggle to staff more frequent screening. More research is needed to determine how social risk and needs evolve naturally to guide healthcare system efforts to intervene upstream.

## Data Availability

The study was part of ongoing quality improvement efforts. As such, it was deemed not human subjects research after administrative review by the KP IRB; informed consent was not required. Because of this quality improvement status, the datasets generated for the current study are not publicly available.

## Abbreviations

**AHC**: Accountable Health Communities
**EHR**: Electronic Health Records
**IRB**: International Review Board
**KP**: Kaiser Permanente
**PABAK**: Prevalence-adjusted Kappa statistic
**RR**: Relative risk
**SDoH**: Social determinants of health
**SONNET**: The Social Needs Network for Evaluation and Translation
